# Pneumomediastinum and Pneumorrhachis Associated With Cannabinoid Hyperemesis Syndrome

**DOI:** 10.7759/cureus.32380

**Published:** 2022-12-10

**Authors:** Laura R Hernandez Garcia, Suzanne Kemper, Shawn A Chillag

**Affiliations:** 1 Medicine, West Virginia University School of Medicine, Charleston, USA; 2 Outcomes Research, Charleston Area Medical Center (CAMC) Health Education and Research Institute, Charleston, USA; 3 Internal Medicine, West Virginia University School of Medicine, Charleston, USA

**Keywords:** marijuana use and hospitalization, pneumorrhachis, spontaneous pneumomediastinum, complications, cannabinoid hyperemesis syndrome

## Abstract

More complications continue to be reported with the increasing use of marijuana (MJ) in the United States, including the increasing prevalence of Cannabinoid Hyperemesis Syndrome (CHS). To our knowledge, based on a thorough review of the literature, we present the third case of CHS with associated pneumomediastinum (PM) and the first case of pneumorrhachis (PR) in a young healthy patient. The main objective of this paper is to heighten awareness of CHS and its potential complications. A brief discussion of a focused history is essential for diagnosis, proper evaluation, and treatment.

## Introduction

As cannabinoid use worldwide continues to rise, cannabinoid hyperemesis syndrome (CHS) is becoming an increasingly seen problem. CHS is a clinical diagnosis, characterized by episodic vomiting that resembles cyclic vomiting syndrome in association with prolonged cannabis use and relief of symptoms after prolonged cessation of marijuana (MJ) use. The use of hot baths or showers to relieve symptoms although not necessary helps support the diagnosis of CHS. Direct and indirect complications of CHS and MJ use have been reported, with pneumomediastinum (PM) rarely reported. A case of recurrent CHS is presented with a discussion of CHS and the first case of PM and pneumorrhachis (PR) in association with CHS. Awareness of these disorders should be heightened to avoid misdiagnoses and unnecessary investigations. For CHS, prevention with discontinuation of MJ is infrequently successful.

## Case presentation

A 23-year-old healthy man was seen at an urgent care facility for three days of nausea, vomiting and abdominal pain with increased bowel sounds and diffuse abdominal tenderness most prominent in the epigastric area. He refused testing at that time and was given omeprazole. Three days earlier he visited another facility with similar symptoms, however, no records were available. Eight hours later, he came to the emergency department (ED) vomiting up to 15 times per day, along with daily, or near daily, use of smoking MJ and tobacco. The last time he used MJ was three days before arriving at the ED; however, the specific amount of MJ used was not described at this time. He reported taking hot showers for some relief of abdominal pain and night-time vomiting. He had no other medical comorbidities, along with no prior surgical history, or current medication use. Vitals were within normal limits (T: 37.8 °C, HR: 67 [Peripheral], HR: 92 [Monitored], RR: 16, SpO_2_: 96%) except for an elevated blood pressure of 149/85. The physical examination was unrevealing.

**Table 1:** Laboratory values during the first ED visit

Laboratory results	Value
WBC	18.6 x10^3^/mcL (High)
Hemoglobin	17.7 g/dL (High)
Sodium	137 mmol/L
Potassium	3.5 mmol/L
Chloride	95 mmol/L (Low)
Total protein	8.6 g/dL (High)
Albumin level	5.5 g/dL (High)
Total bilirubin	3.3 mg/dL (High)
Alkaline phosphatase (ALP)	96 unit/L
ALT	12 unit/L
AST	17 unit/L
Direct bilirubin	0.45 mg/dL (High)
UDS screen	THC positive

**Figure 1:**
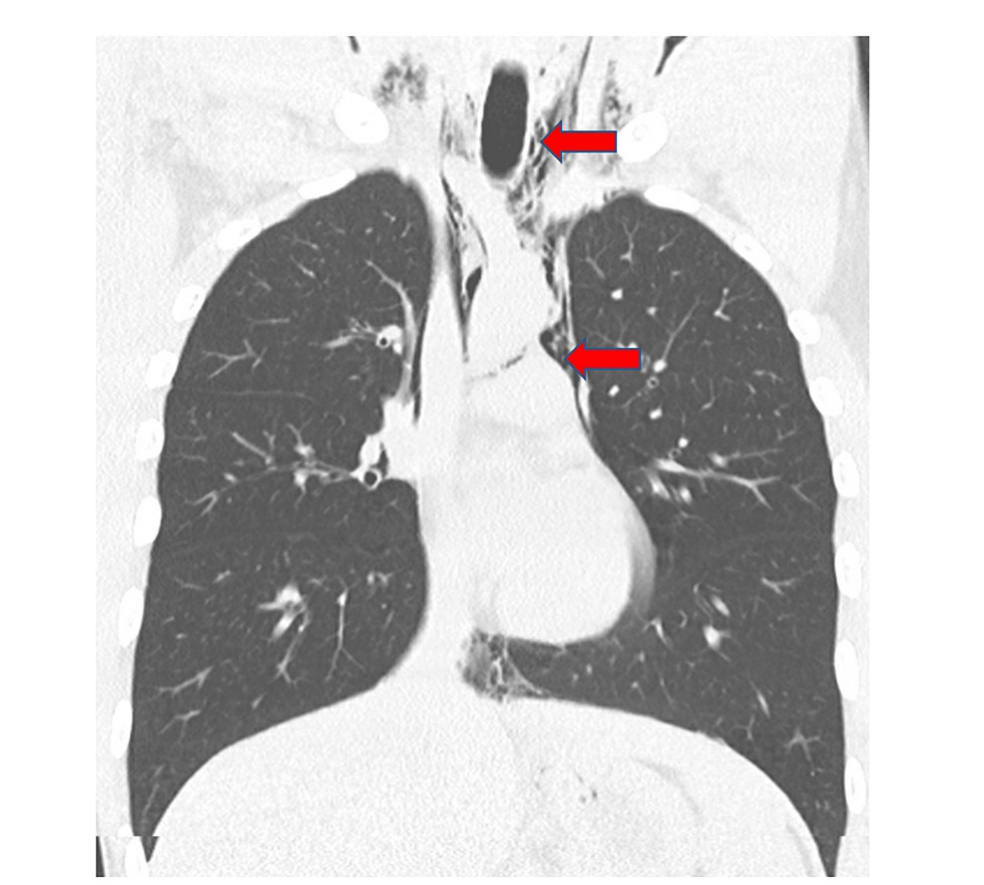
CT chest without contrast showing pneumomediastinum with subcutaneous emphysema in the neck. No pneumothorax was noted.

**Figure 2:**
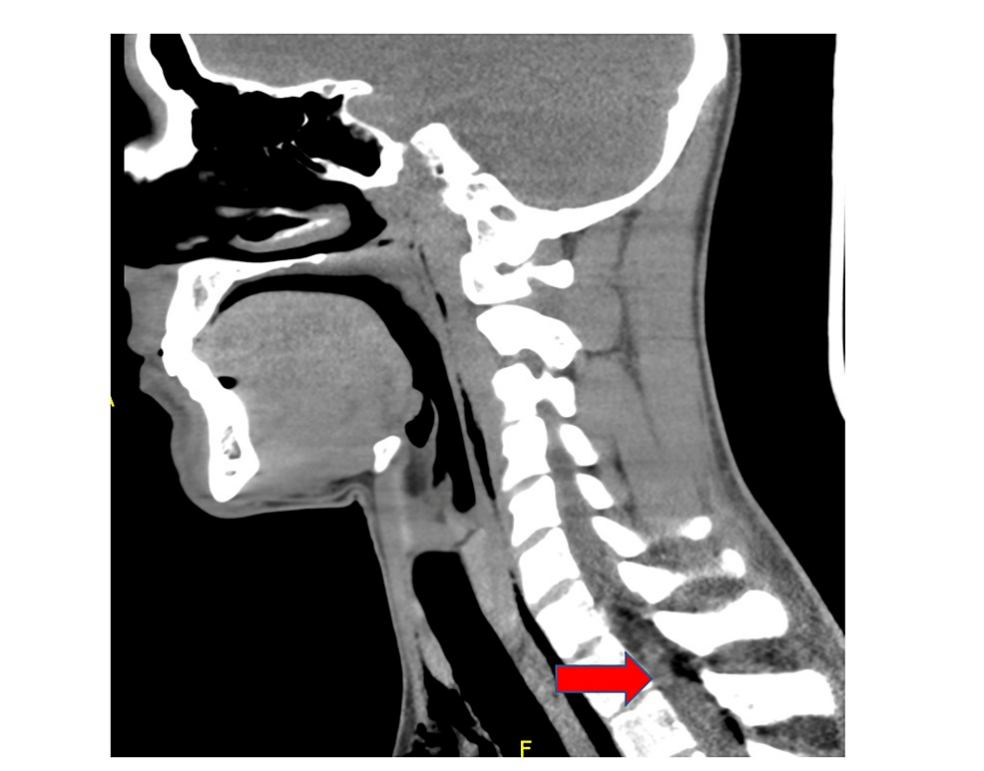
CT soft tissue neck without contrast showing soft tissue emphysema tracking from the mediastinum into the neck and air in the spinal canal (PR).

A chest x-ray revealed PM that was well seen on chest CT, and PR was seen in the neck CT (Figures 1, 2). No other findings were seen on abdominal CT. An esophageal swallowing test did not reveal any leakage. Patient was treated conservatively and given capsaicin topical cream and promethazine as needed for nausea and vomiting. Three days later, the chest x-ray did not reveal the PM; he was discharged to the care of his primary care provider (PCP) with advice to discontinue MJ. It is unclear at this time whether the patient had followed up with his PCP after discharge. It was felt that he may have had a microesophageal perforation without any significant extra-luminal damage or rupture of a peripheral bleb causing the PM and PR.

A year later, he returned to the ED with signs and symptoms like his initial episode. The patient admitted to daily MJ use; however, relief of symptoms with MJ cessation or by taking hot showers/baths was not documented at this time, which would support the CHS diagnosis. Vitals during this admission were unremarkable, and the physical exam showed mild generalized abdominal tenderness but was otherwise normal. Laboratory workup showed markedly elevated white blood cell count (WBC) (Table 2); however, infectious workup including blood cultures and hepatitis panel was negative. The patient had an upper endoscopy during this admission which revealed erythematous gastritis with normal gastric biopsies. The acute kidney injury (AKI) was treated with hydration.

**Table 2:** Laboratory values during second ED visit

Laboratory test:	Value
WBC:	31.5 x10^3/mcL (High)
RBC:	6.32 x10^6/mcl (High)
Hemoglobin:	18 g/dL (High)
Sodium:	137 mmol/L
Potassium:	4.4 mmol/L
Chloride:	100 mmol/L
BUN:	28 mb/dL (High)
Creatinine level:	1.8 mg/dL (High)
Total Protein:	10.1 g/dL (High)
Albumin Level:	6.6 g/dL (High)
Total Bilirubin:	2.2 mg/dL (High)
Alkaline Phosphatase (ALP):	115 unit/L (High)
ALT:	17 unit/L
AST:	19 unit/L
Direct Bilirubin:	0.30 mg/dL (High)
UDS screen:	THC positive

## Discussion

CHS was first described in 2004 and is very similar to cyclic vomiting syndrome except for the association with heavy MJ use and amelioration of symptoms with very hot showers/bathing. Anonymous surveys of patients referred to a cyclic vomiting center who had MJ use showed that 67% of patients had symptom relief with hot showers/baths [1]. This describes how helpful this question is considering CHS as a diagnosis since sometimes patients omit information regarding their MJ use.

A non-clinical survey study identifying 205 with CHS found there may be a genetic predisposition to develop CHS [2]. The mechanism of this emetic response to what is usually a valuable anti-emetic and appetite stimulant is not well understood but may be a disruption of the extensive endocannabinoid system by acting on the CB1 receptors when patients take high doses of THC [3]. This dysregulation may explain why the usual anti-emetic drugs are not very efficacious and better results are seen using anxiolytics, antipsychotics and thermotherapy with topical capsaicin or hydro-thermotherapy, like hot showers [4]. It is believed that thermotherapy relieves symptoms also by the relationship between THC and the thermoregulatory centers in the hypothalamus. Activation of CB1 receptors in the hypothalamic axis can theoretically alter the thermoregulatory sensations initiating the need to take hot showers [5]. Additionally, the only effective prophylactic treatment is the cessation of cannabis use [4].

CHS has been linked to numerous cases of AKI and three suspected deaths [6]. Additionally, only two other cases of otherwise spontaneous PM in association with CHS have been reported [7]. The mechanism of PM seems likely to be a micro-tear in the esophagus that thus far has not been reported to cause Boerhaave syndrome which requires anything beyond observation. Another theory proposed to explain the association of spontaneous PM, and hyperemesis syndrome, includes the increase in thoracic pressure caused by Valsalva maneuvers during vomiting which in turn predisposes an individual to barotrauma that can rupture bullae/blebs [8]. This association of MJ smoking leading to bullae ruptures potentially causing PM and pneumothorax is more likely to be seen with concomitant tobacco smoking [9,10]. Likewise, we believe PR is another potential complication of CHS. Although located in the spinal canal, it has not been reported in association with CHS, but it has been seen in up to 9.5% of cases with PM and often presents with few symptoms specific to the diagnosis [11]. No known cases of PR have been reported with CHS. Although PR and PM are uncommonly reported with CHS, it can be seen in other conditions that cause severe vomiting through the mechanisms mentioned above.

The expectation is CHS may be more common than currently reported. For instance, MJ use is expanding for several reasons including medical usage, recreational use, legalization, availability, lesser stigma with usage, pain management with fewer problems than narcotics, advertising, and less strict enforcement of prohibitive laws. With legalization of recreational MJ in Colorado, there has been an increase in vomiting illnesses by 30% in MJ users presenting to the ED [9]. Unfortunately, there is no mention of CHS on the CDC website among the many adverse effects listed for MJ [12].

## Conclusions

The symptoms and signs of CHS are usually very distressing to patients and perplexing to caregivers who are unaware of the disorder. Heightened awareness of this disorder is paramount for diagnosis, proper treatment and avoidance of unnecessary evaluation and ineffective therapy. Knowledge of the stereotypical presentation of CHS, including improvement of symptoms with hot showers, and history of MJ use can result in early consideration, with additional guided therapy and recommendations. In addition, the only effective treatment is MJ cessation. Substance use counseling and appropriate primary care follow-up are essential in the management of this condition.
